# Rolipram Protects Mice from Gram-negative Bacterium *Escherichia coli*-induced Inflammation and Septic Shock

**DOI:** 10.1038/s41598-019-56899-6

**Published:** 2020-01-13

**Authors:** Xiaying Lu, Juan Wang, Xiaohuan Chen, Yong Jiang, Zhixing K. Pan

**Affiliations:** 1 Department of Pathophysiology and Key Laboratory of Proteomics of Guangdong Province, School of Basic Medical Sciences, Southern Medical University, Guangzhou, China; 2 Department of Medical Microbiology and Immunology, University of Toledo Medical Center, Toledo, Oh 43614 USA

**Keywords:** Bacterial infection, Sepsis

## Abstract

Sepsis is typically triggered by an overwhelming systemic inflammatory response to pathogens, and may lead to severe organ dysfunction and/or death. Sepsis consequently has a high mortality rate and a high rate of complications for survivors, despite modern medical advances. Therefore, drug identification and validation for the treatment of sepsis is of the utmost importance. As a selective phosphodiesterase-4 inhibitor, rolipram also exhibits the abilities of inhibiting multiple pro-inflammatory cytokines production in macrophages and toxin-induced inflammation in mice. However, this drug has never been studied as a sepsis treatment method. We found that rolipram significantly improves survival in mice challenged with gram-negative bacterium *E. coli*, CLP, or *E. coli* derived lipopolysaccharide. We have also found that rolipram inhibits organ damage, pro-inflammatory cytokine production, and intracellular migration of early-stage inflammatory elements. Our results also show that rolipram increases anti-inflammatory cytokine production. The protective effects of rolipram on septic mice may result from inhibition of the MAP kinase and NF-κB signaling pathways. Rolipram may therefore be a potential novel sepsis treatment, one that would bypass the time-consuming and costly drug-discovery process.

## Introduction

Sepsis is triggered by an overwhelming systemic inflammatory response to pathogenic microorganisms, and may lead to multiple organ dysfunction syndrome (MODS) and death. Sepsis is associated with high morbidity and mortality, even under optimal conditions of critical care. It is the leading cause of death in non-coronary intensive care units^1^. The hospital case-fatality rate of severe sepsis patients has been estimated at approximately 25%, while close to 50% of septic shock patients die^2^. Most importantly, the incidence of sepsis has risen by nearly 30% in the past decade^3^. Because sepsis can be induced by different microorganisms, vaccine development is difficult to impossible. Moreover, the abuse of antibiotics in the hospital setting has induced drug resistance in common pathogens. Therefore, despite enormous advancements in understanding the pathogenesis of sepsis, drug treatment remains largely ineffective. The identification, validation, and development of more effective drug treatments is extremely important.

While sepsis can be induced by a number of traumatic and/or pathogenic events, it is commonly induced by a host inflammatory reaction to a number of different microbes and their products^3–6^. Gram-negative bacteria are the leading cause of infection in both hospital-acquired and community-acquired infections. Lipopolysaccharide (LPS) is a major component of the gram-negative bacterial cell wall, and is itself the biggest player in induction of this antibacterial immune response^7^. LPS induces inflammation by binding to Toll-like receptor 4 on the host cell surface. Upon activation of this receptor, the adaptor protein MyD88 is recruited to the receptor, which in turn triggers a cascade of signaling events that leads to the transcription of the transcription factor NF-κB and the mitogen-activated protein (MAP) kinases. A subsequent series of downstream pro-inflammatory reactions leads to the expression of cytokines, chemokines, stress-responsive proteins, and other inflammatory elements^8^.

NF-κB plays a pivotal role in the transcription of pro-inflammatory cytokines, including tumor necrosis factor α (TNF-α), interleukin-1β (IL-1β), and IL-6. MAP kinases, including extracellular signal-related kinase (ERK), c-Jun NH2-terminal kinase (JNK), and p38, also modulate cytokine expression at multiple levels. ERK is required for the transportation of TNF-α mRNA from the nucleus to the cytoplasm^9^. p38 is a critical mediator of the production of pro-inflammatory cytokines, and has been identified as the target of a class of small-molecule inhibitors capable of inhibiting this process^10,11^. LPS-induced transcription and translation of inflammatory mediators leads to inflammatory reactions, organ damage, and ultimately shock in the host^12^.

Rolipram is a typical phosphodiesterase-4 (PDE-4) inhibitor, which can inhibit TNF production in activated mouse macrophages. Furthermore, studies have shown that rolipram markedly restrains carrageenan-induced acute inflammatory responses in mice^13^. Both human and animal experiments have shown that rolipram is also an effective antidepressant, indicating significant bioavailability and effectiveness in the blood^14,15^. However, rolipram has never been studied as a sepsis treatment. We therefore hypothesized, based on preliminary results, that rolipram may protect mice from *E. coli* induced septic shock and *E. coli* derived LPS resultant massive inflammatory response. Our findings indicate that rolipram may protect mice from sepsis and septic shock-like symptoms induced by *E. coli*, CLP, or *E. coli* derived LPS, through inhibition of the NF-κB and MAP kinase signaling pathways. Rolipram is already approved and in use for chronic obstructive pulmonary disorder in the United States. As a result, if rolipram is shown to be a viable sepsis treatment in future experiments, significant time and money that would otherwise be spent on the drug-discovery process may be avoided altogether.

## Results

### Rolipram significantly reduces the mortality rates in multiple mice septic models

**Figure 1:**
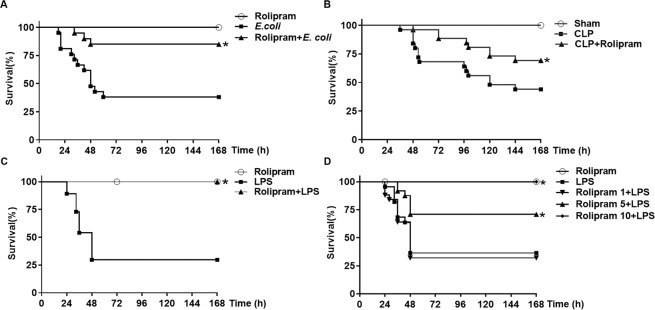
Effects of rolipram treatments on the survival rate (%) of septic shock mice within 7 days. (**A**) Male C57BL/6 mice were injected with rolipram (10 mg/kg, i.p.) 6 hrs before *E. coli* injection (1.5 × 10^8^ CFU, i.p.), rolipram group: n = 10, *E.coli* group: n = 20, rolipram + *E.coli* group: n = 20. (**B**) Male C57BL/6 mice were injected with rolipram (15 mg/kg, i.p.) 6 hrs after CLP surgery, sham group: n = 10, CLP group: n = 25, CLP + rolipram group: n = 26. (**C**) Male C57BL/6 mice were injected with rolipram (10 mg/kg i.p.) 1 hr before LPS injection (15 mg/kg i.p.), rolipram group: n = 10, LPS group: n = 37, rolipram + LPS group: n = 32. (**D**) Male C57BL/6 mice were injected with different doses of rolipram (1 mg/kg, 5 mg/kg, and 10 mg/kg, i.p.) 1 hr before LPS injection. Survival of mice was monitored for 7 days. Kaplan-Meier analysis, followed by a log-rank test, was used for survival time analysis. *Represents p < 0.05 in treatments vs. LPS groups.

To assess the protective effect of rolipram on sepsis induced by *E. coli*, CLP, or *E. coli* derived LPS, we investigated the effect of the drug on survival rate. First, mice were injected intraperitoneally (i.p.) with rolipram or vehicle 6 hr before *E. coli* injection. In the absence of rolipram, 62% of infected mice died within 60 hr of *E. coli* injection (Fig. 1A). In contrast, injection with rolipram resulted in 15% mortality over 7 days, suggesting that rolipram pretreatment can prevent E. coli -induced septic shock in mice. Pretreat the CLP model mice can also significant improve the mice survival rate, from 44% to 69% (Fig. 1B). To confirm the protective effect of rolipram both in E. coli-induced and CLP-induced septic mice, we assessed the role of rolipram in mouse sepsis induced by lipopolysaccharide (LPS) derived from E. coli. Mice were i.p. injected with rolipram 1 hr before LPS injection. In the absence of rolipram, 73% of endotoxic mice died within 48 hr of LPS injection, but 100% mice survival in the rolipram pretreated group (Fig. 1C). These results suggest that rolipram may have a protective effect in sepsis.

The survival dose-response curve for rolipram indicates that the mice receiving the highest dose, 10 mg/kg rolipram, experienced the most benefit (Fig. 1D). Taking into account the differential survival rates and occurrence of first mortality, 10 mg/kg rolipram showed the highest efficacy of all tested concentrations. Rolipram at 5 mg/kg significantly improved the survival rate to 71% as opposed to 36% (p < 0.05). 1 mg/kg rolipram did not significantly improve the survival rate (33%) compared to the LPS-only group.

### Rolipram significantly reduces *E. coli* derived lipopolysaccharide-induced release of serum pro-inflammatory cytokines in mice

**Figure 2:**
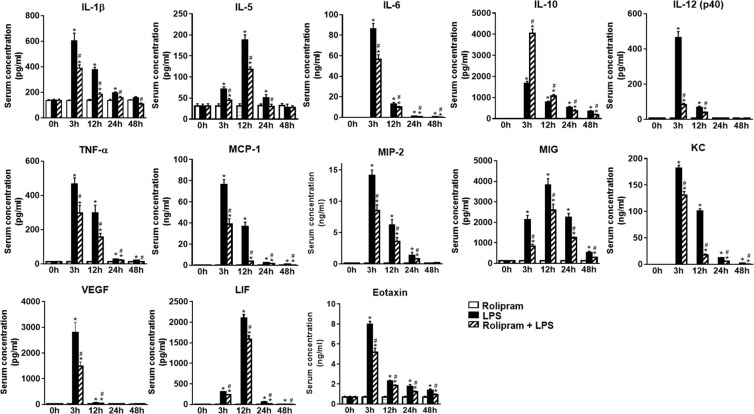
Influence of rolipram on pro-inflammatory cytokines in serum of mice challenged with LPS. Mice received vehicle or rolipram (10 mg/kg i.p.) 1 hr before LPS (15 mg/kg i.p.) injection. Blood samples were collected at 0, 3, 12, 24, and 48 hr after LPS injection for cytokine/chemokine assays. Data was collected from 5 mice in each group and was represent the mean and SD. Comparisons between two groups were performed using two-tailed unpaired Student’s T-tests. *Represents p < 0.05 vs. control, ^#^represents p < 0.05 vs. LPS alone.

The overwhelming release of pro-inflammatory cytokines plays an important role in the pathology of sepsis. Therefore, the serum levels of multiple pro-inflammatory cytokines were examined. Results show that the concentrations of IL-1β, IL-5, IL-6, IL-12 (p40), TNF-α, MCP-1, MIP-2, eotaxin, KC, MIG, LIF, and VEGF, as well as the anti-inflammatory cytokine IL-10, were all significantly elevated in serum after 3 hr and 12 hr of LPS challenge (Fig. 2). In contrast, administration of rolipram effectively reduced the production of pro-inflammatory cytokines and chemokines, and further increased the levels of IL-10.

### Rolipram prevents *E. coli* derived lipopolysaccharide-induced lung injury in mice

**Figure 3:**
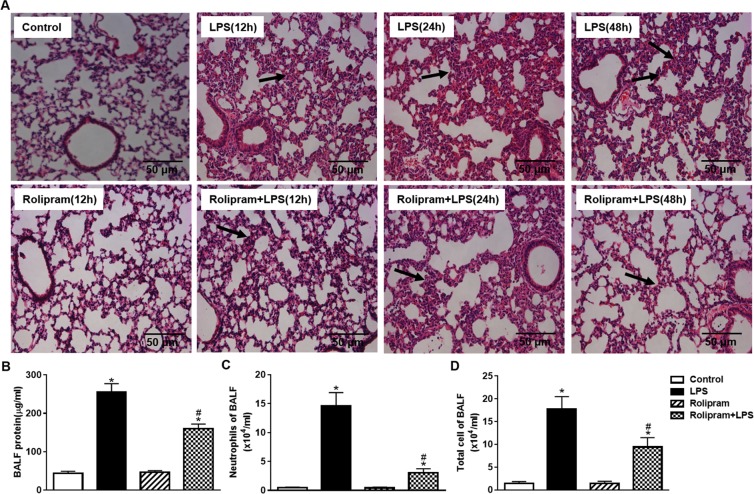
Effects of rolipram on LPS-induced lung injury. (**A**) Representative photographs of lung tissues stained with H&E, original magnification x 20. Mice received vehicle or rolipram (10 mg/kg i.p.) 1 hr after LPS (15 mg/kg i.p.) injection. Lungs were collected at the indicated time points post injection. (**B**) BALF protein concentration, (**C**) BALF neutrophil count, and (**D**) total BALF cell count increases were significantly attenuated by rolipram. Lungs were lavaged at 24 hr before LPS injection for BALF assays. Data was collected from 5 mice in each group and was represent the mean and SD. Comparisons between two groups were performed using two-tailed unpaired Student’s T-tests. *Represents p < 0.05 vs. control, ^#^represents p < 0.05 vs. LPS alone.

LPS-induced endotoxic shock is known to cause a number of effects in the murine host, including severe lung injury. Histopathological analysis, consisting of hematoxylin and eosin (H&E) staining of lung sections from LPS-only mice, revealed signs of extreme inflammation. Edema, neutrophil recruitment, and hemorrhage in the lung samples were also seen (Fig. 3A). Lung injury in the LPS group was visibly increased in comparison with the control group. Rolipram treatment, however, markedly attenuated LPS-induced lung injury. In bronchiolar lavage fluid, total protein (Fig. 3B), absolute neutrophil count (Fig. 3C), and total cells count (Fig. 3D) were significantly reduced.

### Rolipram alleviates *E. coli* derived lipopolysaccharide-induced liver and kidney damage

**Figure 4:**
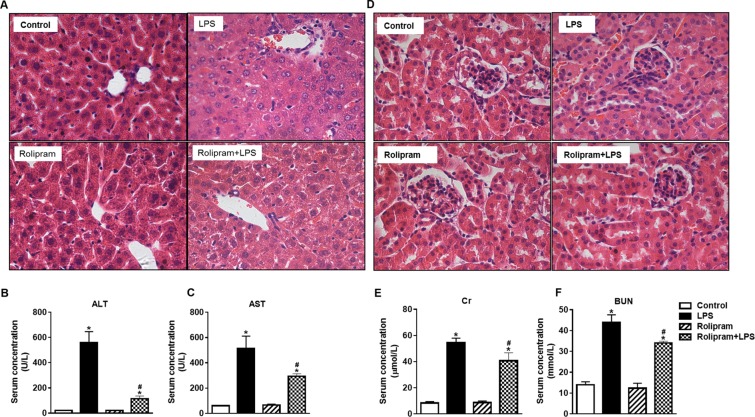
rolipram alleviates liver and kidney damage caused by LPS. (**A**) Liver tissue of mice shows normal hepatic lobule and space anatomy. LPS injection caused loss of normal anatomy; however, rolipram treatment ameliorated these changes. (**B**) Rolipram prevents LPS-induced kidney injury. (**C**–**F**) Biochemical markers for liver and kidney injury are significantly reduced in rolipram-injected mice as compared to LPS-only positive controls. Blood samples and organs were collected at 24 hr after LPS injection. Data represent mean and SD of at least three independent experiments, performed in triplicate. Comparisons between two groups were performed using two-tailed unpaired Student’s T-tests. *Represents p < 0.05 vs. control, ^#^represents p < 0.05 vs. LPS alone.

H&E staining of liver tissue from control mice showed that the liver plate was radially arranged around the central vein in normal hepatic lobules. The livers of LPS-only mice sacrificed at the 24-hr time point showed structural disorder of the hepatic lobule, narrowing or even disappearance of the hepatic sinusoidal space, vacuolar degeneration of hepatocytes, and nuclear pyknosis. However, rolipram pre-treatment for 1 hr significantly reduced these changes (Fig. 4A). Renal tissue H&E staining (Fig. 4B) clearly showed normal structure and morphology in glomeruli, renal tubules, and renal tubular epithelial cells in control mice. The renal lumen was regular, and no inflammatory cells infiltrated the renal interstitial cells. The kidneys of LPS-only mice at the 24-hr time point showed inflammatory infiltrate in the renal interstitial cells, swelling of renal tubular epithelial cells, and an unclear cell gap. Rolipram pretreatment for 1 hr significantly reduced these changes. We also examined biochemical markers for liver and kidney injury in serum from each group of mice treated with LPS for 24 hr. Rolipram inhibited levels of aspartate and alanine aminotransferases (AST and ALT), creatinine (Cr), and brain natriuretic peptide (BUN) in serum. This indicates that rolipram can ameliorate liver and kidney injury induced by LPS (Fig. 4C–F).

### Rolipram inhibits *E. coli* derived lipopolysaccharide-induced activation of the NF-κB and MAPK signaling pathways in mouse lungs

**Figure 5:**
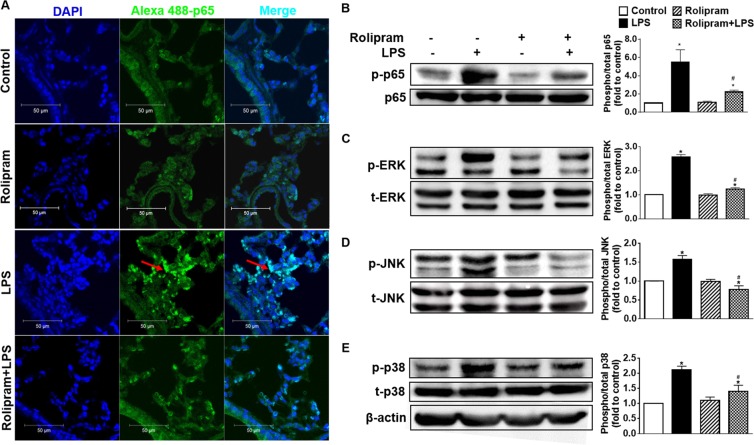
Effects of rolipram on activation of the NF-κB pathway and MAPK in mouse lung tissue. (**A**) Rolipram inhibits LPS-induced NF-κB/p65 nuclear translocation in lung tissue. Mice received vehicle or rolipram (10 mg/kg i.p.) 1 hr after LPS (15 mg/kg i.p.) injected. Lungs were collected for immunofluorescence staining 3 hr after LPS injection. (**B**) Rolipram decreased LPS-induced NF-κB/p65 phosphorylation in lung tissue. Mice received vehicle or rolipram (10 mg/kg i.p.) 1 hr after LPS (15 mg/kg i.p.) injection. Lung tissues were collected 3 hr after injection for Western blot. The phosphorylated NF-κB/p65 chemiluminescent signal was quantified and normalized to total NF-κB/p65. Phosphorylated NF-κB/p65 is expressed in arbitrary units. LPS-stimulated cells were set as 100%, and other values are taken from said setting. (**C**–**E**) Mice received vehicle or rolipram (10 mg/kg i.p.) 1 hr before LPS (15 mg/kg i.p.) injection. Lungs were collected at 3 hr after injection. The chemiluminescent signal was quantified; phosphorylated ERK, JNK, and p38 MAPK were normalized against total ERK, JNK, and p38 MAPK. Phosphorylated protein levels are expressed in arbitrary units. LPS-stimulated cells were set at 100%, and other values relate to that setting. Data represent the mean and SD of at least three independent experiments, performed in triplicate. Significant differences between more than two groups were performed using ANOVA. Comparisons between two groups were performed using two-tailed unpaired Student’s T-tests. *Represents p < 0.05 vs. control, ^#^represents p < 0.05 vs. LPS alone.

Immunofluorescence microscopy using anti-NF-κB/p65 antibody, revealed that LPS induces NF-κB/p65 nuclear translocation in the mouse lung 3 hr after injection. However, rolipram markedly inhibited this translocation (Fig. 5A). Three hours after LPS treatment, phosphorylation of NF-κB/p65 significantly increased in the mouse lung as compared to controls. Rolipram markedly suppressed phosphorylation of this molecule (Fig. 5B). Western blot results showed that LPS treatment induced the phosphorylation of ERK1/2, JNK, and p38 MAPK (Fig. 5B–D). Rolipram significantly suppressed the phosphorylation of all three molecules in mouse lungs 3 hr after administration.

## Discussion

We demonstrate that rolipram may protect against LPS-induced inflammation and shock in mice, likely through the inhibition of the NF-κB and MAPK signaling pathways. We found that rolipram significantly improved animal survival, decreased inflammatory cytokines essential to the process of shock, alleviated organ injury, and dephosphorylated critical inflammatory pathways. These results indicate that rolipram suppresses inflammatory responses that are essential to the process of sepsis, and thus may be protective against sepsis. Therefore, rolipram may serve as a novel drug treatment for inflammatory disease, such as septic shock.

Sepsis is induced by a dysregulated innate immune reaction, leading to a harmful host response to pathogens. This is includes excessive amounts of pro-inflammatory cytokines, such as TNF-α and IL-1β^16,17^, as well as other inflammatory mediators. These molecules can in turn trigger secondary inflammatory processes, leading to inflammatory pathology and life-threatening organ damage. Proteins that induce the migration of inflammatory cells into tissue are also upregulated. We have found that rolipram inhibits pro-inflammatory cytokines and chemokines released by the administration of LPS, leading to the suppression of excessive inflammatory responses, cell adhesion and migration, and the further sequelae of shock and multiple organ failure in the mouse host. Sepsis-induced multi-organ dysfunction and injury are the main mechanisms of patient shock and death. Therefore, sepsis treatment guidelines consider multi-organ dysfunction a key point of attack in sepsis treatment^18^. It has previously been reported that rolipram significantly decreased hyperoxia-induced neutrophil numbers in BALF and inhibits IL-6 and MCP-1 transcription in rat lungs^19^. Rolipram also alleviates pulmonary edema and reduces neutrophil numbers in BALF during chlorine-induced mouse shock^20^. Our results go further in showing that rolipram inhibits pulmonary edema, neutrophil infiltration in alveoli, and protein concentrations and neutrophil numbers in BALF. Liver and kidney function is also improved, indicating that rolipram can prevent LPS-induced multi-organ failure. The exact mechanism by which rolipram controls the inflammatory cascade, thereby preventing shock and multiple organ failure in the host, is unknown. However, it is known that the NF-κB pathway is crucial in the regulation of inflammatory gene expression. Nuclear translocation activates the transcription and expression of a variety of cytokines and adhesion molecules, all of which are closely associated with inflammation and the immune response^21^. The NF-κB family, including p65 (RelA), p50/p105 (NF-κB1), p52/p100 (NF-κB2), RelB, and c-Rel, exist in the cytoplasm in homo- or heterodimers that can bind with IκB. During conditions of stimulation by LPS or other pro-inflammatory factors, IκB kinase (IKK) phosphorylates IκB, leading to NF-κB nuclear translocation and further activation of inflammatory genes^22^. It has been confirmed that toll-like receptor 4 -NFκB signaling plays a key role in acute lung injury^23^. Our studies show that LPS activates NF-κB and its signaling pathways, while rolipram restrains activation. This indicates that the NF-κB may be part of the mechanism by which rolipram exerts its effects.

The MAPK signaling pathway also plays a critical role in regulating inflammation and the immune response. As a conserved signaling cascade, the MAPK pathway exists in almost all eukaryotic cells. MAPKs can regulate target protein function through phosphorylation, thus participating in cell proliferation, growth, differentiation, and function. MAPK signaling is activated through three levels, including MAP3K, MAPKK, and MAPK. At present, four MAPK signaling pathways have been identified: ERK1/2, ERK5, p38 MAPK, and JNK^24^. It has been found that rolipram promotes the maturation of glial progenitor cells and regeneration of myelin through enhancement of ERK phosphorylation^25^. Studies have also found that rolipram restrains bone cancer pain through inhibition of the JNK signaling pathway in the bone marrow, suppressing neuron-stellate cell activation^26^. In addition, rolipram can inhibit LPS-induced p38 MAPK phosphorylation in J774 cells^13^. However, the role of rolipram on phosphorylation of the MAPK downstream signaling pathways has not yet been reported. Our results indicated that rolipram suppresses LPS-induced ERK, JNK, and p38 MAPK phosphorylation in lung tissue, suggesting that the MAPK signaling pathway – as well as NF-κB – may mediate the anti-inflammatory actions of rolipram.

In conclusion, we have found that rolipram protects mice from massive inflammatory responses and endotoxic shock through inhibition of NF-κB and MAPK signaling pathway activation. Rolipram may thus serve as a novel drug treatment for inflammatory disease, namely sepsis and septic shock. As rolipram is an approved drug in the United States for chronic obstructive pulmonary disorder, this drug repositioning strategy may circumvent the lengthy and expensive drug discovery process, and allow faster and better treatment for sepsis patients.

## Methods

### Reagents

*Escherichia coli* 0127:B8 was obtained from ATCC (12740). LPS (*Escherichia coli* 0127:B8) was purchased from Sigma-Aldrich (St. Louis, MO, USA). Rolipram was obtained from Enzo Life Science (Farmingdale, NY, USA). Rabbit anti-p38, anti-phospho-p38, anti-ERK1/2, anti-phospho-ERK1/2, anti-JNK, anti-phospho-JNK, and horseradish peroxidase (HRP)-conjugated goat anti-rabbit IgG were procured from Bioworld Technology (St. Louis, MN, USA). Rabbit anti-NFκB p65 and anti-phospho-NFκB p65 antibodies were obtained from Cell Signaling Technology (Danvers, MA, USA). Alexa Fluor® 488 goat anti-rabbit IgG (H + L) was purchased from Molecular Probes (Eugene, OR, USA). MILLIPLEX® MAP Mouse Cytokine/Chemokine Magnetic Bead Panel Kit for 96-Well Plate Assay was purchased from EMD Millipore (Darmstadt, Germany).

### Animals

Male C57BL/6 mice, weighing 20–25 g, all 8–10 weeks old, were used for experiments. The mice were purchased from the Department of Laboratory Animal Science of Southern Medical University (Guangzhou, China). All animals were housed with free access to food and water under conditions of optimal light, temperature, and humidity (12:12-hr light-dark cycle, approximately 24 degrees Celsius, 50–60% humidity). All experimental procedures were approved by the Ethics Committee of Animal Research at the College of Medicine, Southern Medical University, and were conducted in accordance with the international guidelines for care and use of laboratory animals.

### Animal model

Lipopolysaccharide was dissolved in phosphate-buffered saline (PBS) and stored at −20 degrees Celsius. Rolipram was dissolved in dimethyl sulfoxide (DMSO) and further diluted in PBS (final DMSO concentration: <0.05%). First, male C57BL/6 mice were intraperitoneally (i.p.) injected with 200 µL rolipram (10 mg/kg) 1 hr before, 1 hr after, or at the same time as LPS (15 mg/kg in 200 µL). Survival rate was monitored for 7 days. The experiments were repeated using variable doses of rolipram (1, 5, and 10 mg/kg). At 0, 3, 12, 24, and 48 hr after LPS injection, mice were anesthetized and sacrificed. Blood was collected for analysis of serum cytokines. For histological analysis, normal saline (10 mL) was perfused through the right heart ventricle. Lung, liver, and kidney samples were collected and processed as described later. Mouse blood samples were left at room temperature for 1 hr, then spun at 1000 × g for 15 minutes at 4 degrees C. Serum was obtained and stored at −80 degrees C until required.

### Cytokine assays

Cytokines from mouse serum were measured using the MILLIPLEX® MAP Mouse Cytokine/Chemokine Magnetic Bead Panel Kit for 96-Well Plate Assay, run on a Luminex platform. Cytokines include the three primary cytokines of interest: tumor necrosis factor-α (TNF-α), interleukin-1-β (IL-1β), and IL-6. Multiple cytokines were also explored: IL-1α, IL-2, IL-5, IL-7, IL-9, IL-10, IL-12(p40), IL-12(p70), IL-13, IL-15, IL-17, IFN-γ inducible protein 10 (IP-10), eotaxin, interferon-γ (IFN-γ), monocyte chemoattractant protein-1 (MCP-1), macrophage inflammatory protein-1α (MIP-1α), MIP-1β, MIP-2, migration-inducing protein (MIG), keratinocyte chemoattractant (KC), leukemia inhibitory factor (LIF), lipopolysaccharide-induced CXC chemokine (LIX), lipopolysaccharide-induced CXC chemokine (RANTGES), granulocyte-macrophage colony-stimulating factor (GM-CSF), G-CSF, M-CSF, and vascular endothelial growth factor (VEGF). For quality assurance, each sample was run twice.

### Liver and renal function tests

Serum activity of aspartate aminotransferase (AST, a nonspecific marker for hepatic injury), alanine aminotransferase (ALT, a specific marker for hepatic parenchymal injury), blood urea nitrogen (BUN, a marker for glomerular renal function), and creatinine (Cr, a marker for glomerular filtration rate and renal failure) were measured by Fengrui Biotechnology Company kits (Hunan, China).

### Broncheoalveolar lavage (BAL) fluid collection and analysis

BAL was performed immediately after 24 hr of LPS challenge. Lungs were lavaged with PBS (0.5 mL × 3). The recovery rate of BAL fluid (BALF) was approximately 80%. Cells were isolated from the BALF by centrifugation of 500 x g for 5 minutes, and the cell pellets were resuspended. The total cells in the BALF were counted using a hemocytometer. Subsequently, the cell suspension was cytospin-centrifuged onto microscope slides and stained using Liu Stain (Baso, China). The percentage of neutrophils was determined by counting 400 cells. The BALF supernatant was analysed for total protein concentration by using bicinchonic acid (BCA) assay, according to the manufacturer’s instructions.

### Histology

Lungs, livers, and kidneys were fixed in 4% paraformaldehyde (pH 7.4). The organs were then dehydrated and embedded in paraffin. Sections 5 µM thick were cut. The slides were then stained with hematoxylin and eosin (H&E), and examined by a light microscope.

### Western blot analysis

Total protein was extracted from the frozen lung tissue using T-PER Tissue Protein Extraction Reagent (Pierce, IL, USA). Protein concentrations were determined using a BCA Assay Kit (KeyGEN, China). Equal amounts of lung tissue protein (100 µg per animal) were run on a 10% SDS-PAGE gel and transferred onto polyvinylidene difluoride (PVDF) membranes. The membranes were blocked with 5% bovine serum albumin (BSA) in TBST at room temperature for 2 hr, and then incubated with primary antibody against mouse ERK1/2, p-ERK1/2, JNK, p-JNK, p38, p-p38, or p65 (all at a 1:1000 antibody:BSA ratio) at 4 degrees C overnight. After 3 washes with TBST, the membranes were incubated in secondary HRP-conjugated anti-rabbit IgG at room temperature for 1 hr. The membranes were then washed with TBST, processed with an ECL detection kit (Pierce, IL, USA), and measured on film in a darkroom.

### Immunofluorescence

All tissue stainings were performed on frozen 8 µM-thick OCT-embedded mouse lung tissue sections. Sections were blocked to eliminate non-specific binding with 0.5% BSA in PBS for 1 hr, then incubated with primary antibody against mouse phosphorylated p65 (1:100) at 4 degrees overnight. After subsequent washes with PBS, the sections were incubated with Alexa Fluor® 488 goat anti-rabbit IgG (H + L) for 1 hr at 37 degrees C, then washed with PBS. The slides were stained with DAPI (Santa Cruz Biotechnology, CA, USA) for 5 min. Slides were then washed in PBS. Coverslips were mounted onto the slides using anti-fade reagent (Beyotime Biotechnology, China). The images were acquired using a Zeiss LSM 710 confocal microscope (Zeiss, Germany).

### Statistical analysis

Data are expressed as the mean plus or minus standard deviations. Statistical evaluation was performed with SPSS software (Version 20.0). Significant differences between more than two groups were performed using ANOVA. Comparisons between two groups were performed using two-tailed unpaired Student’s T-tests. Kaplan-Meier analysis, followed by a log-rank test, was used for survival time analysis. P < 0.05 was taken as the maximum significant difference.
